# The stigma of obesity in the general public and its implications for public health - a systematic review

**DOI:** 10.1186/1471-2458-11-661

**Published:** 2011-08-23

**Authors:** Claudia Sikorski, Melanie Luppa, Marie Kaiser, Heide Glaesmer, Georg Schomerus, Hans-Helmut König, Steffi G Riedel-Heller

**Affiliations:** 1 Leipzig University Medical Center, IFB AdiposityDiseases, Leipzig, Stephanstraße 9c, 04103 Leipzig, Germany; 2 Institute of Social Medicine, Occupational Health and Public Health, University of Leipzig, Philipp-Rosenthal-Str. 55, 04103 Leipzig, Germany; 3 Department of Medical Psychology and Medical Sociology, University of Leipzig, Philipp-Rosenthal-Str. 55, 04103 Leipzig, Germany; 4 Department of Psychiatry and Psychotherapy, University of Greifswald, Rostocker Chaussee 70, 18437 Stralsund, Germany; 5 Department of Medical Sociology, Social Medicine and Health Economics, Hamburg-Eppendorf University Medical Center, Martinistr. 52, 20246 Hamburg, Germany

## Abstract

**Background:**

Up to this date, prevalence rates of obesity are still rising. Aside from co-morbid diseases, perceived discrimination and stigmatization leads to worsen outcomes in obese individuals. Higher stigmatizing attitudes towards obese individuals may also result in less support of preventive and interventive measures. In light of the immense burden of obesity on health care systems and also on the individuals' quality of life, accepted and subsidized preventive measures are needed. Policy support might be determined by views of the lay public on causes of obesity and resulting weight stigma. This study seeks to answer how representative samples of the lay public perceive people with obesity or overweight status (stigmatizing attitudes); what these samples attribute obesity to (causal attribution) and what types of interventions are supported by the lay public and which factors determine that support (prevention support).

**Methods:**

A systematic literature search was conducted. All studies of representative samples reporting results on (a) stigmatizing attitudes towards overweight and obese individuals, (b) causal beliefs and (c) prevention support were included.

**Results:**

Only 7 articles were found. One study reported prevalence rates of stigmatizing attitudes. About a quarter of the population in Germany displayed definite stigmatizing attitudes. Other studies reported causal attributions. While external influences on weight are considered as well, it seems that internal factors are rated to be of higher importance. Across the studies found, regulative prevention is supported by about half of the population, while childhood prevention has highest approval rates. Results on sociodemographic determinants differ substantially.

**Conclusions:**

Further research on public attitudes toward and perception of overweight and obesity is urgently needed to depict the prevailing degree of stigmatization. Introducing a multidimensional concept of the etiology of obesity to the lay public might be a starting point in stigma reduction.

## Background

Public awareness of obesity has changed substantially. During the early 2000s only 2 to 3 per cent of the population considered obesity to be one of the most important health issues [1], while nowadays the majority in e.g. Germany recognizes the significance of the problem [2]. Despite this rise in awareness and willingness to accept obesity as a chronic condition of clinical significance, obese individuals are subject to a high level of stigmatization resulting in discrimination [3]. A recent review by Puhl & Heuer (2009) finds disadvantages for obese people in numerous areas, including employment, health care settings as well as in interpersonal relationship aspects [4].

Discrimination is seen as a resulting phenomenon which is based on negative attributes. Therefore, every evidence of existing discrimination also supports the existence of negative attribution. Stigma as proposed by Jones et al. (1984), in elaboration of Goffman's definition, is a "mark" that links a person to undesirable characteristics [5,6]. Hence, the terms of negative attribution and stigmatizing attitudes are used to describe the same mechanism. Being a prequel of following discrimination, the nature of these attitudes needs to be investigated.

Attribution theory provides the theoretical framework for why negative attributes are ascribed to obese individuals [7]. For obesity, the negativity of attributes can be explained by the influence of causal beliefs and responsibility. DeJong showed in experiments that both play a central role in negative attribution [8,9]. Crandall & Moriarty conclude from their study that the more a disease is perceived as under volitional control, the more it is stigmatizing - with obesity generally being perceived as highly under control [10,11]. A further study on a number of health problems including obesity found perceptions of level of severity and behavioral causation of these conditions to predict greater social rejection [7]. Corrigan (2003) provides an attribution model of public discrimination. In this model, causal beliefs about the controllability of the condition lead to an emotional response (e.g. stigmatization attitudes). Behavioral consequences in the form of discrimination result [12].

While a recent review summarizes discrimination and stigmatizing attitudes [4], so far, causal beliefs on obesity have not been summarized in a comprehensive review yet. Negative attributes include labeling obese individuals as lazy, unintelligent and unmotivated [3,4,13]. Translated into public policy support, higher stigmatizing attitudes may result in less support of preventive and interventive measures. Since these stigmatizing attitudes might be based on causal attribution to the individual, the public might not see the need or justification to support and finance efficient prevention measures. In light of the immense burden of obesity on health care systems [14,15], prevention efforts that are accepted and potentially subsidized by the public are crucial to obviate a further rise in obesity prevalence rates.

Furthermore, Puhl and Heuer (2010) show that perceived weight stigma and discrimination have a vast impact on the quality of life of overweight individuals, including higher probabilities to show unhealthy eating and activity behavior [16,17]. Indeed, it could also be assumed that weight discrimination influences treatment rates and help-seeking behavior for weight reduction opportunities. Together, these factors provide basis for a further rise in obesity prevalence rates [4]. Respectively, comprehensive knowledge on these components of weight stigma will further help to evaluate existing models of stigmatization as well as promoting the development of new models.

For all components - stigmatizing attitudes, causal attribution and prevention support - research has mainly been based on samples in selected settings, e.g. students [18,19]. This study seeks to answer (a) how representative samples of the lay public perceive people with obesity or overweight status (stigmatizing attitudes); (b) what these samples attribute obesity to (causal attribution) and (c) what types of interventions are supported by the lay public and which factors determine that support (prevention support).

## Methods

### Literature search

This review was prepared according to the systematic literature review guidelines of the Centre for Reviews and Dissemination [20] and follows PRISMA (Preferred Reporting Items for Systematic reviews and Meta-Analyses) suggestions [21]. A systematic literature search available on the electronic databases Medline, Web of Science, PSYNDEXplus, EMBASE and Cochrane Library was conducted in February 2011. The terms (obes* OR adiposity* OR overweight* OR over-weight* OR fat) AND (attitude* OR belief* OR prejudice* OR stigma* OR perception*) AND representative served as search criteria. Additional File 1 shows the Medline search strategy in detail. In addition, the bibliographies of the selected articles were searched.

### Inclusion criteria

Abstracts were screened by two authors using the following selection criteria: (i) nationally or community-based representative studies (ii) of the adult (≥ 18 years) general population and (iii) reporting on attitudes towards, stereotypes of, or the perception of overweight and obese people as an outcome variable.

### Data extraction

Titles and abstracts were screened to identify studies of likely relevance and full papers obtained. Primarily, methodical data on sampling, study design, explored constructs, and definition of outcome criteria were extracted from all selected studies. Secondly, the selection criteria described in the above section were then reapplied to ensure accurate study inclusion.

## Results

### Study characteristics

**Figure 1:**
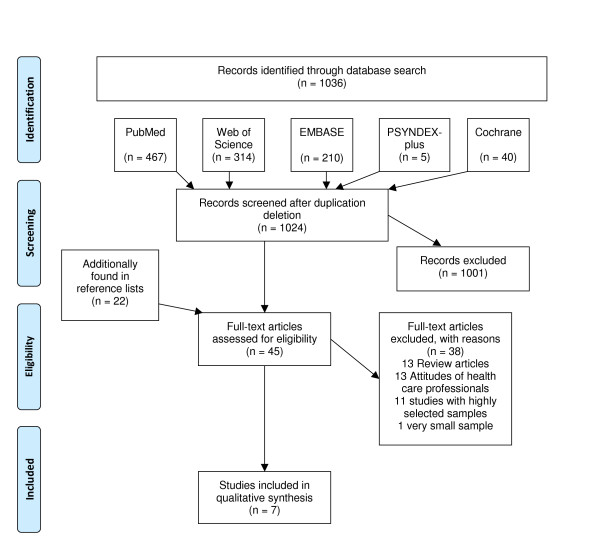
**Search Strategy**. Search terms: (obes* OR adiposity* OR overweight* OR over-weight* OR fat) AND (attitude* OR belief* OR prejudice* OR stigma* OR perception*) AND representative.

The results of the systematic literature search are shown in Figure 1. Initially, 1024 articles were found in the search. From those, 45 potentially relevant articles were identified after screening of abstracts. Twenty-two of these were found in reference lists of the identified articles. After retrieving all full articles, 38 further articles were rejected as not fulfilling the selection criteria. Seven articles were assessed and included for detailed analysis. Table 1 gives an overview on study characteristics and used measures. Three articles were based on the same study and will be reported as one. Most studies found in the process of literature search investigated the opinion of the U.S. population [22-25]. There was only one representative German survey [2,26,27]. Sample sizes varied from N = 909 to N = 2,250. All selected studies surveyed nationally representative samples of individuals aged 18 years and older, with a mean age of 45.9 years.

**Table 1:** Study Characteristics

Study	n	Sample Description	Age	Survey Method	Construct covered and main measurements used
[22] Barry, Brescoll, Brownell & Schlesinger (2009); USA	1009	nationally representative web sample RDD-sampling to recruit for web sample; *Yale Rudd Center Public Opinion on Obesity Survey*	≥ 18 yrs	Internet survey	**Causal attribution **described in 7 specific metaphors: obesity as sinful behaviour; a disability; a form of eating disorder; a food addiction; a reflection of time crunch; a consequence of manipulation by commercial interests; as result of a toxic food environment For what percentage of overweight Americans does [metaphor] account for? **Policy support **(7 redistributive, 6 compensatory, 3 price-raising): Rating of support

[2,26,27] Hilbert, Rief, Brähler, 2007a, 2007b; 2008 Germany	1000	nationally representative ADM-sampling with last birthday method; surveyed by USUMA	45.9 yrs	Telephone interview - structured interview - CATI - 20 min	**Causal attribution **conceptualized in 11 items: behavioural, other environmental, genetic risk factors → rating on a 5-point Likert scale. **Policy support **(11, information-based campaigns, regulatory measures and childhood-focused measures): Rating of support **Stigmatizing attitudes**: Subscale "weight/control blame" (WCB) of the Antifat Attitudes Test. **Causal attribution: **3 scales covering behaviour, environment and heredity

[23] Oliver & Lee, 2005; USA	909	nationally representative; RDD-sampling; *American Attitudes towards Obesity (AATO) survey*	/	Telephone interview	**Causal attribution**: 2 items each on 3 dimensions (genetic, environmental, personal attribute factors) **Policy support **(regulating food ads and lunches in school): Rating of support on 5-point Likert scale

[24] Seo, Torabi & Torabi, 2006; USA	1000	nationally representative; RDD-sampling	≥ 18 yrs	Telephone interview - CATI - 10 min	**Causal attribution: **2 items ("Obese people can do sth. about their weight" and "Obese people can lose weight by watching their eating habits")→ rating on 5-point Likert scale.

[25] Taylor, Funk, Craighill, 2006; USA	2250	randomly-selected nationally representative	≥ 18 yrs	Telephone interview	**Causal attribution**: Rating of reasons of overweight/obesity

**Abbreviations: **ADM --- Arbeitsgemeinschaft Deutscher Marktforschungsinstitute (German specific three stage random sampling method); CATI - Computer-Assisted Telephone Interview; RDD - Random Digit Dialing.

The instruments applied in the investigations were primarily constructed by the authors themselves and were based on previous research and current literature. One group used metaphors derived from elite discourse and previous research [22,28]. Another applied the subscale "Weight Control/Blame" (WCB) from the Antifat Attitude Test [27,29]. Overall, three out of five studies recruited their participants through Random Digit Dialing-sampling [22-24]. The majority of research teams conducted their investigation via telephone interviews. One applied an internet survey procedure [22].

### (a) Stigmatizing attitudes

Only one article reports explicit measures of stigmatizing attitudes. Hilbert et al. (2008) found an average WCB score of 3.01 (scale range: 1 = strongly disagree to 5 = strongly agree) [27]. A mean score of 3 indicated mainly neutral answers. In an analysis of response patterns, the authors categorized 23.5% of all respondents as displaying definite stigmatizing attitudes (WCB score ≥ 3.50) while 21.5% showed no stigmatizing attitudes (WCB score ≤ 2.49). Entered into a regression equation with stigmatizing attitudes as the dependent variable, causal attribution of obesity to behavior (internal) contributed to the explanation of variance the most (r^2 ^= 0.10). Further variables predicting higher stigmatizing attitudes were less education, not seeing obesity as an illness, older age and fewer causal attribution of obesity to heredity. Those five variables accounted for a total of 18% the variance.

### (b) Causes of obesity/causal attribution

In an earlier publication of the same study, Hilbert et al. report results of agreement with different perceived causes of obesity that were allocated to underlying factors [2]. The most prevailing causal attributions were lack of activity behavior (82.4%) and overeating (72.8%). External factors were rated less important - only 34.9% agreed on heredity to be important, and, respectively, only 23.6% of all respondents found the lack of activity environment to be of importance. However, about half of the respondents (53.8%) agreed a bad food environment to be one possible cause of obesity. Men were less likely to report causal attribution to the food environment while attribution to activity behavior was found to be associated with lower income (leading to less attribution to activity) and higher age (higher agreement rates).

Two items of the study by Seo et al. (2006) can be regarded as proxy measures of causal attribution. The assumptions that obese individuals can do something about their weight and lose weight by watching their eating habits are causes ascribed to the individual, e.g. personal attribution. For each item, almost three quarters of the respondents agreed. Being of Hispanic decent (item: "can do sth. about their weight", compared to black and white ethnicity) and an education level of some college (item: "watch eating habits", compared to High School level) showed to be demographic correlates leading to lower agreement rates [24]. Another study by Oliver & Lee (2005) finds an attribution to lack of willpower to be agreed on most often (65% agree or strongly agree). The authors assessed agreement with two items on each factor of possible explanations of obesity (genetics, environmental and personal attribute). Agreement with environmental factors was highest on average (59.5%), followed by personal attribute (55%) and genetic influences (29%).

Reasons under control of the individual rank highest in the study by Taylor et al. (2006) - not getting enough exercise (75%) and lack of willpower (59%) are seen as more important causes than food environment (50%) and genetics (32%). As for demographic correlates, higher percentages of women describe the reasons "lack of exercise" and "food marketing" as very important. Blacks and Hispanics are inclined to put slightly more emphasis on genetic factors, still ranking it lowest, as Whites do.

In context of the totally different approach used by Barry et al. (2009) by assessing perceived causes of obesity with metaphors, both, metaphors displaying high individual blame (obesity as a sinful behavior, an addiction) and metaphors with low individual blame (industry manipulation, toxic food environment) are seen as important or very important explanations [22]. Table 2 summarizes results and provides an overview.

**Table 2:** Perceived causes of obesity and demographic correlates

Cause	**Hilbert et al**. [2,26,27]	**Seo et al**. [24]	**Oliver & Lee **[23]^1^	**Taylor et al**. [25]	**Barry et al**. [22]
Lack of activity behavior	82.4 Low income ↓ Higher age ↑	-	-	75 Female gender ↑	-

Overeating	72.8	72.5 Some college ↑	-	-	-

Genetics	34.9	-	29	32 Blacks/Hispanics ↑	-

Lack of activity environment	23.6	-	-	-	-

Bad food environment	53.8 Female gender ↑	-	62	50 Female gender ↑	23.9^2^

Lack of willpower	-	-	65	59	-

Agreement rates in %; sociodemographic factors are listed, arrows indicate higher (↑) or lower (↓) agreement with the corresponding cause; ^1 ^2 items each result in the differentiation of genetic, environmental and internal factors; for reasons of comparability single item results are reported; ^2^ toxic food environment.

### (c) Prevention support

Hilbert et al. assessed support of three categories of prevention efforts (information, regulation and childhood prevention). Support for preventive measures was highest for childhood prevention and informational campaigns (89.7% and 82.2%), while regulative prevention was only agreed on by 42.4% of the participants. Determinants of prevention support were analyzed in multiple linear regression analysis. Attribution of obesity to be a result of the food environment contributed to variance explanation the most. Higher age, female gender and residence in the eastern part of Germany were sociodemographic correlates of prevention support. Furthermore, a greater perceived significance of obesity, stronger societal responsibility for a solution to the obesity problem, and more causal attribution of obesity to lack of activity behavior showed to be significant associations of prevention support [2]. Oliver & Lee (2005) concentrated their survey on preventive measures of the regulative spectrum (food ads, taxing, junk food in schools, the same as in the study by Hilbert et al.). Support was highest for regulating food ads (57% agree or strongly agree), while only 33% of the respondents agreed on taxing snack foods [23]. Overall agreement with regulation corresponds to the results of the study by Hilbert and colleagues [26] - 45.6% show approval of this kind of prevention effort. Older age proved to be an influencing factor: Sixty-five-year-olds are more likely to support all three policies than eighteen-year-olds. High family income predicted opposition to obesity policies. Effects of gender, educational background and ethnicity are mixed across the three items. One item - linking obesity to access to poor foods - showed to be a highly significant predictor for support of all three policies. This result was also found by Hilbert and colleagues [2]. Barry et al. (2009) divided prevention policies into three groups: redistributive (tax increasing), compensatory (helping or protecting citizens), and price-raising policies [22]. These are not commensurate with the categorization by Hilbert et al. and Oliver & Lee; however, it is possible to contrast approval rates for the three policies used in all studies, all of the regulative spectrum (see table 3). Again, highest approval rates are found for TV advertisement regulation and abolishment of junk food in schools while taxing junk food was unpopular. The overall agreement rate is congruent with the other studies (45%). Since demographic correlates are only reported for the three main categories and adequate comparisons are not possible, the authors refrain from reporting these.

**Table 3:** Prevention policy support

Policy	**Hilbert et al**. [2,26,27]	**Oliver & Lee **[23]	**Barry et al**. [22 ]
Restricting advertisement for unhealthy food	47.7	57	52.5

Raising taxes on unhealthy food	26.7	33	28.4

Banning unhealthy food in schools	52.8	47	54.3

Total	42.4	45.6	45

Correlates	Higher age ↑ Female gender ↑ Eastern part of Germany ↑ Attribution to bad food environment and lack of activity behavior ↑	Higher age ↑ Higher income ↓ Attribution to bad food environment ↑	-

Agreement rates in %; sociodemographic factors are listed, arrows indicate higher (↑) or lower (↓) support of preventive measures in general.

## Discussion

This study aimed at reviewing a) prevalence of stigmatizing attitudes, b) causal attribution of obesity of the lay public and its predictors as well as c) determinants of prevention support. Regarding causal attribution as a potential origin of stigmatizing attitudes towards obesity, this review shows that causes that are within the individual's control are named most frequent in population surveys and yield high agreement rates. It seems, however, that the public acknowledges the multicausality of obesity to some extent. Bad food environment in particular is named an important cause by about half of the population in Germany and in the USA. Also, the rated importance of genetics coincides. Research shows that aside from the significant role of genetic and biological factors [30], social and economic variables have to be considered. Exemplarily, Finkelstein and colleagues summarize that, while reduced energy expenditure at the workplace and increased leisure activities equal out, calorie intake has risen in the past 20 years [31]. The Centers for Disease Control and Prevention (CDC) reports a rise in energy intake of 7 to 20 per cent (men/women) since the late 70 s [32], to name just a few societal life-style related factors. Especially women seem to acknowledge this circumstance, seeing the food environment as an important contributor to the obesity problem. This might be a result of an increased awareness for nutritional aspects in general. A German study on nutrition showed women to be overrepresented in healthy nutrition clusters and underrepresented in a cluster describing fast food oriented consumers [33].

However, the differentiation of internal and external attribution is somewhat questionable. Previous analysis of causal attribution showed that attributions to the environment were significantly associated with behavioral attributions and might therefore be assumed to be within the individual's control [27]. Further research on causal beliefs of the lay public is therefore needed. The allocation of causes to internal and external factors will need to be clarified.

Despite considering external influences on weight as well, it seems that internal factors are rated to be of higher importance. Preference of internal factors might be influenced by media coverage. Coverage on obesity emphasizes internal, controllable factors of the condition while neglecting societal contributions [34]. A recent study replicates these results for Germany. Hilbert & Ried quantitatively analyzed national and local newspapers and concluded that the current way of reporting might contribute to weight stigma [35]. On a theoretical level, being perceived as a somewhat voluntary condition, the societal function of obesity stigma can be explained by a model of Phelan and colleagues [36]. The authors propose that the obvious failure to comply with societal norms (and that being the goal of attractiveness and fitness) is expanded to a judgment of morality and character (e.g. lack of willpower). This then leads to "reintegrative shaming" (e.g. stigmatization) and represents an attempt to increase the conformity with the existing norms (attractiveness and fitness). Therefore, stigmatizing obese individuals may motivate them to engage in healthier life style, assuming that individuals will alter behavior to avoid obvious social deviation and the resulting stigmatization [37]. Research challenges that assumption, presenting numerous results of negative consequences of weight stigma [38]. Puhl and Heuer (2010) review a number of studies showing that perceived stigmatization and discrimination results in unhealthy eating behavior, potential eating disorders and lower levels of physical activity, all leading way to further weight gain [16]. As this review shows, prevalence of stigmatizing attitudes is rather high. About a quarter of the German population displays stigmatizing attitudes towards the obese.

On the societal side of stigma consequence, according to attribution theory [7], the attribution of obesity to internal factors leads to negative reactions and less empathy and willingness to help the affected individual. One indicator of such an association might be that this review shows highest support rates for childhood prevention but lowest rates for an increase in taxes and other regulative measures. This circumstance can be regarded as a willingness to support measures that do not influence or restrict the entire society (as it would be with tax rises) but only those that show more of an ideational effect. The result that higher stigmatizing attitudes lead to a higher support of prevention efforts, but less willingness to pay for these, goes in line with that assumption. Also, higher support was associated with higher age. One could argue that tax burden decreases with aging and retirement and therefore, again, a measure is supported that one does not have to pay for directly.

Initially, however, these results are contrary to attribution theory prediction, especially in the light of the factors associated with prevention support. Linking obesity to a bad food environment which, as mentioned before, might be a factor associated with internal control, positively predicts prevention support. The enforcement of social norms as an essential function of stigma in conditions perceived as voluntary, might explain this link more adequate and might be a plausible consequence of stigmatization [36]. Used to increase conformity with norms it seems logic that stigmatization also leads to higher prevention support.

## Limitations

The number of studies that the authors were able to include is limited. Only one study reporting the prevalence of stigmatizing attitudes in a representative sample was found. Especially the review of sociodemographic influences on prevalence of stigmatizing attitudes, causal attribution and prevention support was restricted by the scarce number of studies. Furthermore, the use of different measures to determine stigma makes comparisons rather difficult. Some studies used somewhat validated scales, while another derived metaphors from elite discourse, only allowing for careful validation assumptions.

## Conclusions

This review shows that reliable, population-based studies on the stigma of obesity are not yet sufficient in number and comparability. This is, however, the first review to focus on nationally representative studies. Since obesity is a widespread condition, representative research is needed in order to come to reliable conclusions.

Attribution of obesity to internal causes still seems a major source of stigmatization and discrimination of obese individuals which provides an ideal starting point for intervention approaches: Introducing a multidimensional concept of the etiology of obesity to the public ought to help reduce stigmatization. Such a concept is proposed by Sharma & Padwal. The authors declare obesity to be a sign of underlying causes that lead a positive energy intake balance. They call for an analysis of those underlying - mainly external - factors that contribute to overeating and reduced activity behavior [39].

### Future Perspectives and Practical Implications

Further research on public attitudes toward and perception of overweight and obesity is urgently needed to depict the prevailing degree of stigmatization for several reasons. Data on whom to target with anti-stigma campaigns is lacking. There might be parts of the population that display higher stigmatizing attitudes and thus should be addressed preferably in order to raise policy support. Additionally, obese men and women might experience a different degree of stigmatization. The same might be true for different age groups among the obese.

We suggest an increased use of standardized instruments (also concerning self-stigmatization such as the Inventory of Stigmatizing Experiences [40]) and accordingly focus on the development of such. As for options of actively dealing with stigmatizing attitudes, prevention programs with information campaigns might have a high potential in increasing the awareness on the topic and have shown to be widely accepted. Furthermore, two approaches to stigma reduction and therefore better outcomes of overweight people have arisen - one being the urgent need for modification of prejudice among the general public and thus an effort of reducing weight discrimination, the other being effective coping strategies for the individuals themselves, easing effects of perceived weight discrimination. A return to normal weight is improbable for most obese individuals since most weight-loss interventions only yield a weight loss of about 10 per cent [41]. Stigma and attitude modification therefore play a central role and hold the potential in helping to prevent negative outcomes for affected individuals.

## Competing interests

The authors declare that they have no competing interests.

## Authors' contributions

CS, ML and SRH outlined and specified the research questions. The principal author and MK conducted the literature search and screened abstracts and titles. Article inclusion was also evaluated by MK and SRH. CS wrote the first draft of the manuscript. ML, HHK, HG and GS revised it crititically for important intellectual content. All authors contributed to and have approved the final manuscript.

## Pre-publication history

The pre-publication history for this paper can be accessed here:

http://www.biomedcentral.com/1471-2458/11/661/prepub

## Supplementary Material

Additional file 1 **Search terms for Medline**. Details on the search strategy for Medline. Click here for file
