# Supplementary material for: The stigma of obesity in the general public and its implications for public health - a systematic review
Source: BMC Public Health. 2011 Aug 23;11:661. doi: 10.1186/1471-2458-11-661 (PMC3175190; doi:10.1186/1471-2458-11-661)
Supplement: Additional file 1 — Search terms for Medline. Details on the search strategy for Medline. [file 1471-2458-11-661-S1.DOC]

**Additional File 1 Search terms for Medline**

1. obes*/
2. adiposity*/
3. overweight*/
4. over-weight*/
5. fat/
6. or/(1-5)
7. attitude*/
8. belief*/
9. prejudice*/
10. stigma*/
11. perception*/
12. or/(7-11)
13. representative
14. 6 and 12 and 13
